# Supplementary material for: Double stranded RNA sensing is silenced during early embryonic development
Source: Nat Commun. 2025 Dec 11;16:11438. doi: 10.1038/s41467-025-66352-0 (PMC12749073; doi:10.1038/s41467-025-66352-0)
Supplement: Supplementary file 1 — Supplementary Information [file 41467_2025_66352_MOESM1_ESM.pdf]

Supplementary Figure 1

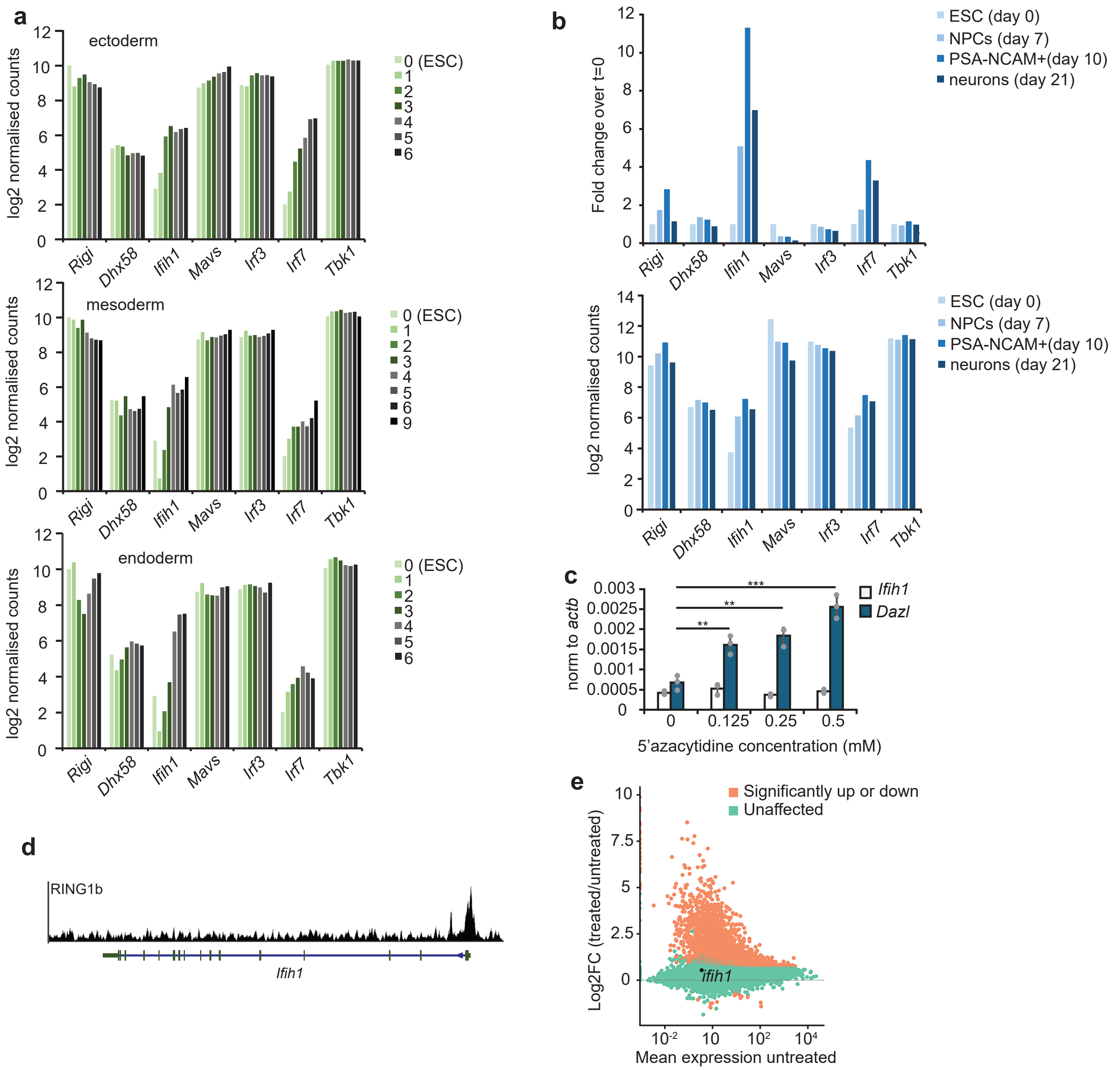

Supplementary Figure S1. *Ifih1* expression is induced upon differentiation. (**a**) Absolute expression levels of genes involved in TLR signalling, RLR signalling pathway and JAK/STAT signalling analysed by high-throughput RNA sequencing of ESCs differentiating to ectoderm (top), mesoderm (middle) and endoderm (bottom) (MTAB_4904, **1**). Samples were taken at successive days after starting differentiation (day 0 corresponds to ESCs, until day 6 or 9 of differentiation). Expression is calculated as log2 normalised counts. (**b**) Relative (top) and absolute (bottom) expression levels of genes involved in RLR signalling in dataset of high-throughput RNA sequencing (GSE125413, **2**). Cells were differentiated from ESC to neurons, with samples taken at days 0 (ESC), day 7 (neural progenitor cells, NPCs), day 10 (PSA-NCAM+ cells) and day 21 (mature neurons). PSA-NCAM is a marker for intermediate differentiation. Expression is calculated as normalised counts relative to ESCs (day 0) for the upper panel and as log2 normalised counts for the lower panel. (**c**) RT-qPCR analyses of *Ifih1* and *Dazl* expression after 5-azacytidine treatment of ESCs. Data are the average of three biological replicates ± SEM, Single factor ANOVA was used to calculate significant differences amongst comparisons, followed by an F-test for variance and Tukey HSD (*) p-val≤0.05, (**) p-val≤0.01, (***) p-val≤0.001. (**d**) Genomic snapshot of RING1B binding at the *Ifih1* gene in ESCs (**3**). (**e**) Mean normalised expression vs expression changes after 8 h of Auxin treatment (log2FC) from nuclear RNA-seq in *Ring1A*-/- AID-RING1B ESCs (66). *Ifih1* is highlighted in black and does not display significant changes in expression.

Supplementary figure 2

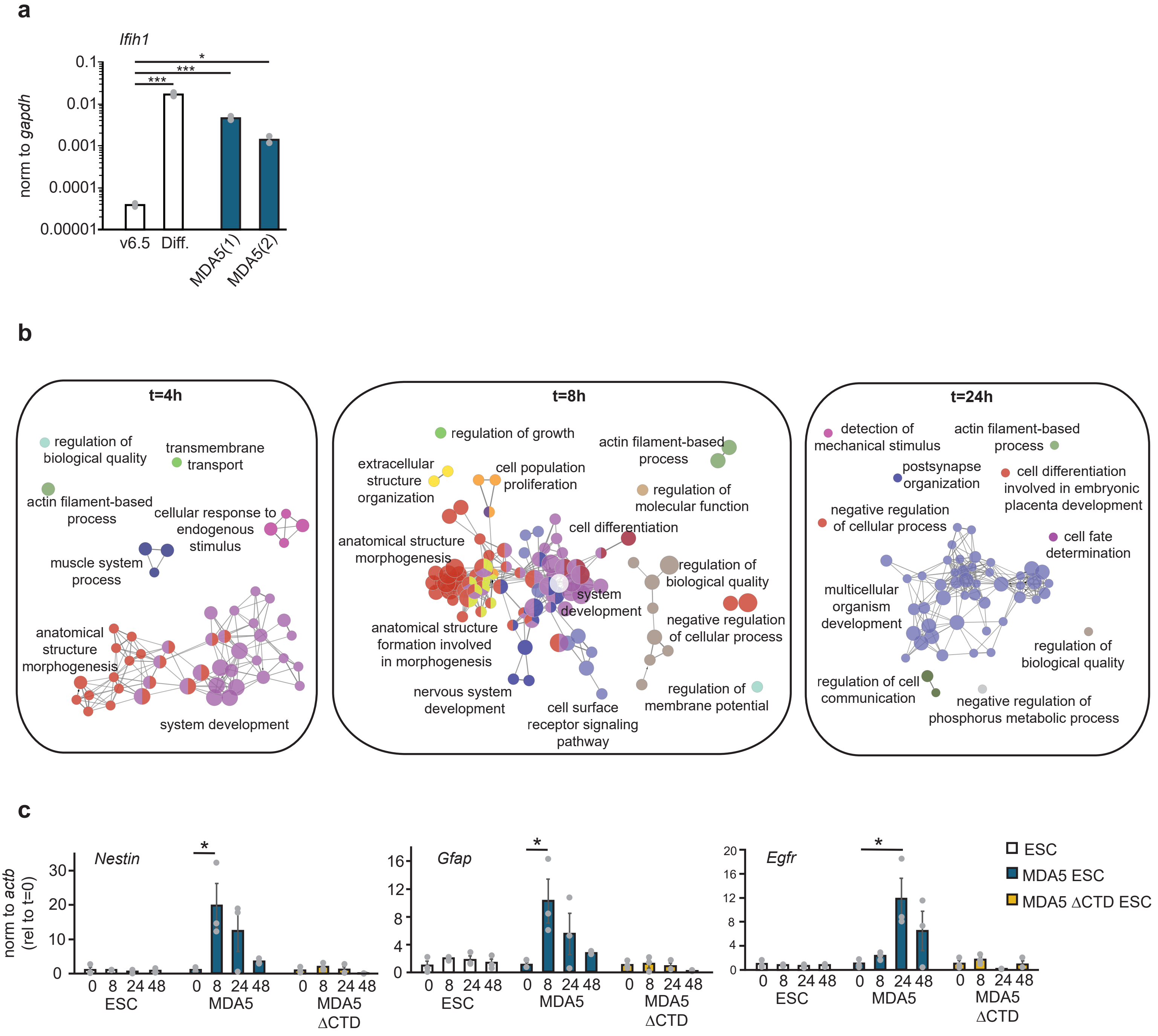

Supplementary Figure 2. Time-course analyses during *Ifih1* induction by RNA-seq. (**a**) *Ifih1* expression in ESCs (v6.5), differentiated v6.5 (Diff) and two v6.5 clones overexpressing MDA5 (MDA5(1) and MDA5(2)). Data are the average of three biological replicates ± SEM for v6.5 and Diff and two biological replicates for MDA5(1) and MDA5(2), Single factor ANOVA was used to calculate significant differences amongst comparisons, followed by Tukey HSD post-hoc test (*) p-val≤0.05, (**) p-val≤0.01, (***) p-val≤0.001.(**b**) Differentially expressed genes (abs log2FC>0.4, p-val≤0.05) that were either up- or down regulated in both clones were used for gene ontology analyses (biological process). (**c**) RT-qPCR analyses of neuronal marker genes in WT ESCs (white) vs ESCs overexpressing WT MDA5 (blue) and its mutant form (ΔCTD, yellow). Samples were taken at 0, 8, 24 and 48 hours after doxycycline addition. Data are the average of three biological replicates ± SEM, Single factor ANOVA was used to calculate significant differences amongst comparisons, followed by Dunnett's post-hoc test (*) p-val≤0.05, (**) p-val≤0.01, (***) p-val≤0.001.

Supplementary Figure 3 - 1

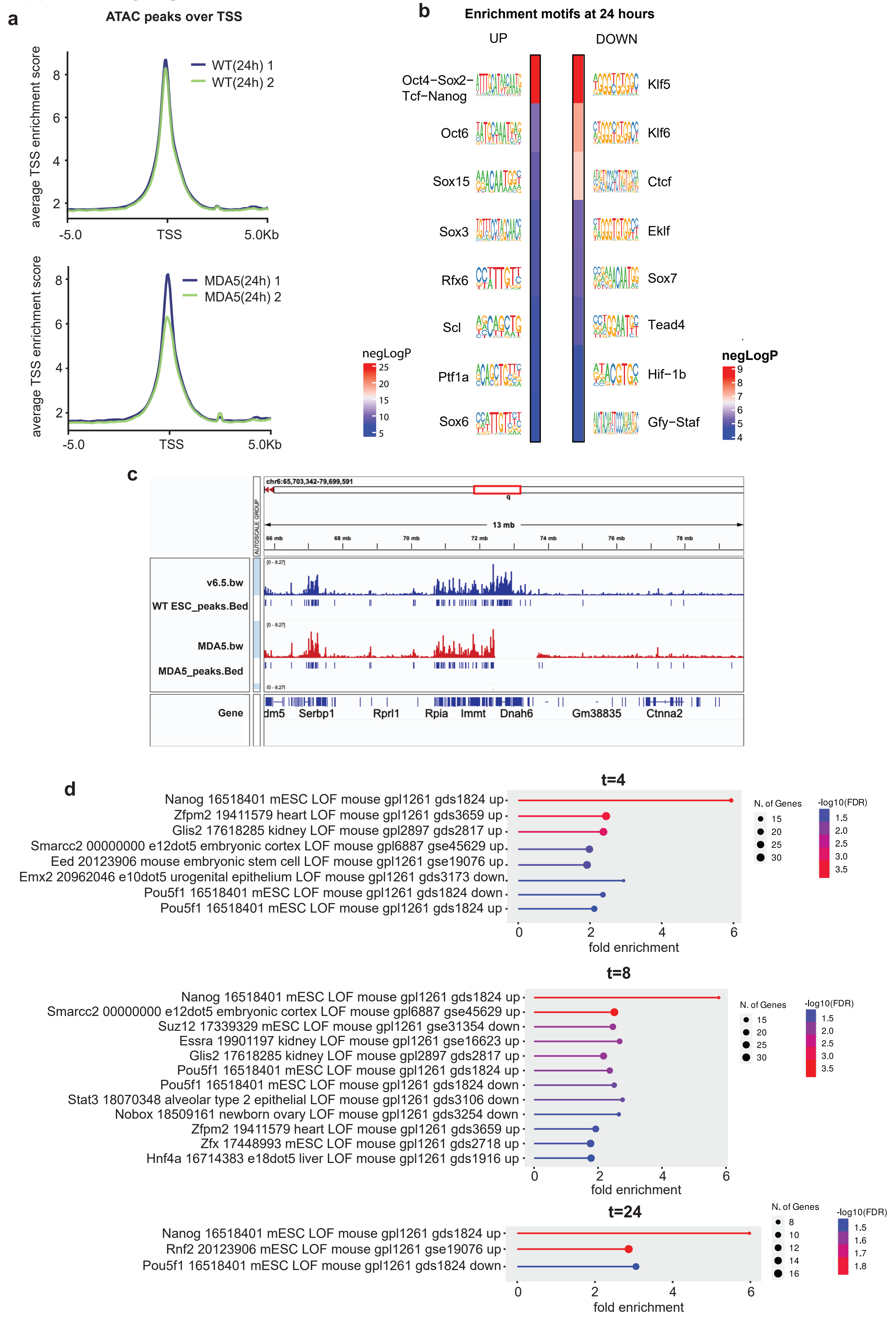

Supplementary Figure 3 - 2

e

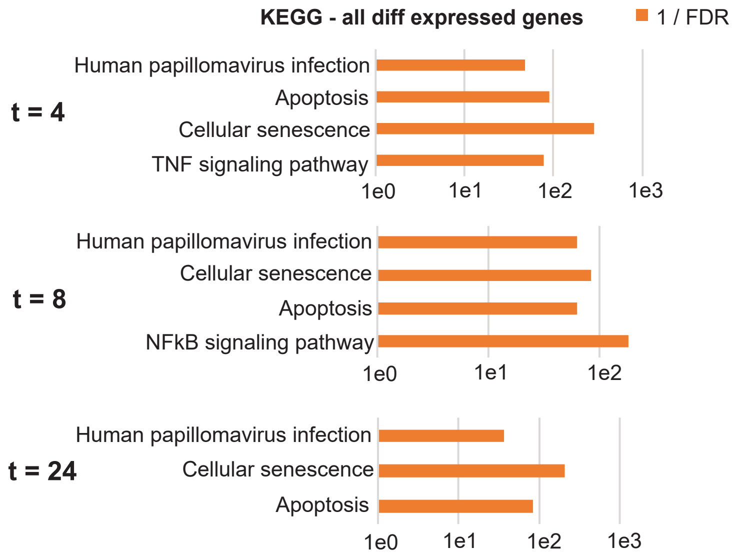

f

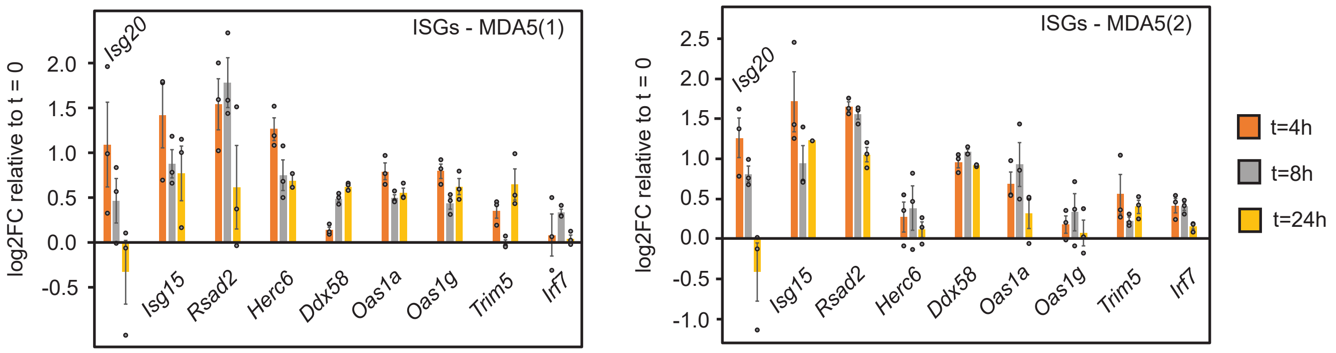

Supplementary Figure 3. ATACseq peaks distribution, motif enrichment and immune activation (**a**) Feature distribution of ATAC peaks with mean read count frequency of peaks at the transcription start site (TSS) for the different experimental samples in WT (top) and MDA5-expressing ESCs (bottom). (**b**) Known motif analysis enrichment on up- and down-regulated ATAC peaks after 24 hours of induction, compared to the initial timepoint (t=0h). Sequence weight matrixes of matched DNA-binding motifs are shown, with -log2(P value) represented by colour. (**c**) ATAC seq analyses revealed a small genomic region absent in the MDA5 clone (1). Comparison with the ATAC-seq data of parental cell line (v6.5) suggests that microdeletion was caused by random insertion of doxycycline-inducible MDA5. The deletion was also confirmed in the RNA-seq data, as no expression of *Dnah6* was detected. The MDA5 clone (2) mESCs display expression for *Dnah6* gene, suggesting that clone (2) does not harbour the same deletion. (**d**) Loss-of-function (LOF) prediction for TFs using the differential gene expression at t=4, 8 and 24h after Ifih1 induction. (**e**) Selected KEGG terms associated with infection and immune responses for all differentially expressed genes in both MDA5 clones at 4-, 8- and 24-hours post-doxycycline treatment. (**f**) Log2FC expression levels of ISGs upon MDA5 overexpression in three biological replicates of clones 1 and 2 by RNA-seq.

Supplementary Figure 4

a

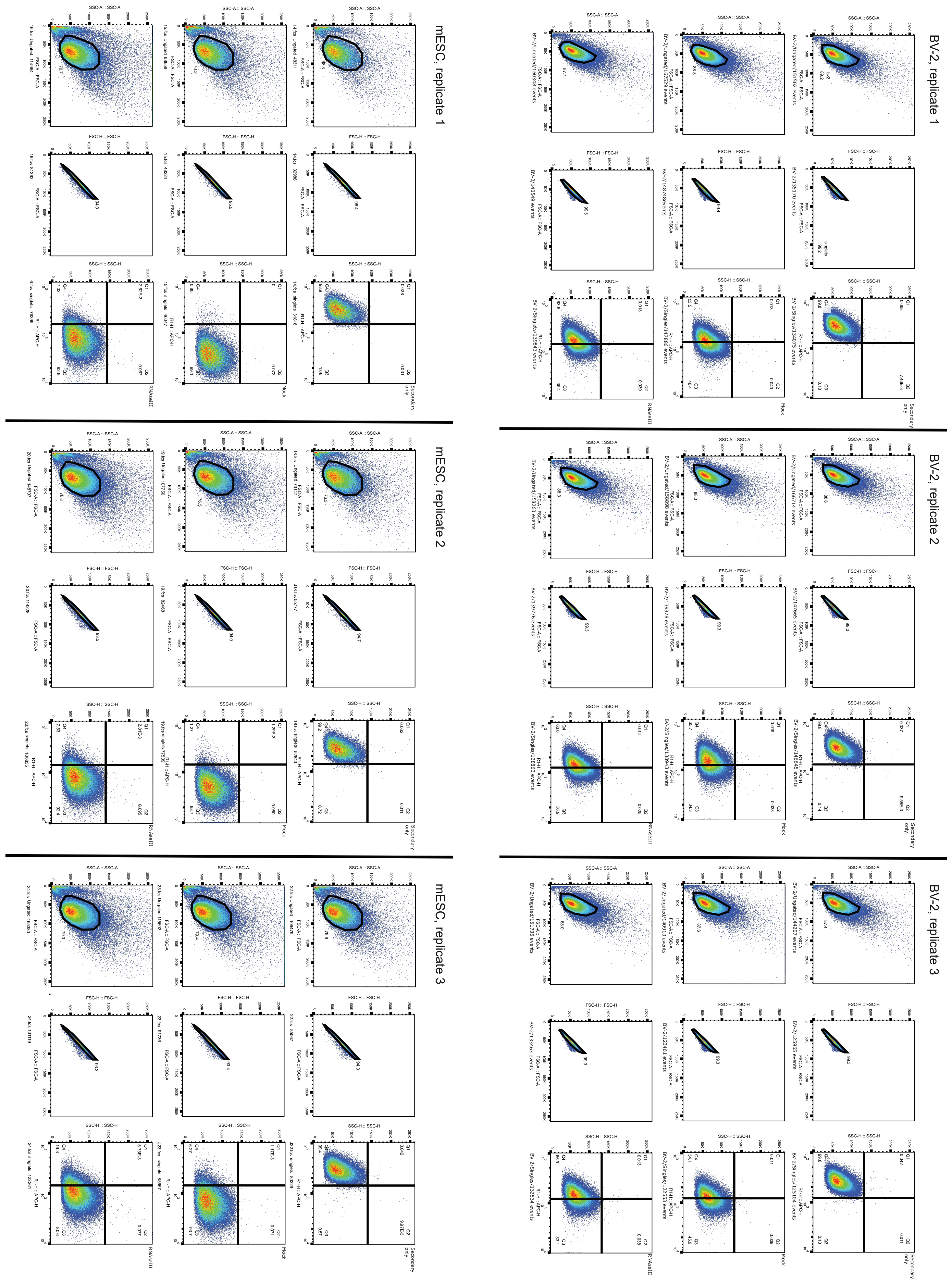

Supplementary Figure 4. Flow cytometry for dsRNA in mouse ESCs and BV2 cells. (**a**) Flow cytometry of three mouse ESCs and BV2 replicates. Black border indicates gated cells from from main population of cells (SSC-A/FSC-A, left panel) and gating for singlets (FSH-H/FSC-A, middle panel). Singlets are used to measure dsRNA signal using the appropriate channel (SSC-H/APC-H, right panel).

Supplementary Figure 5

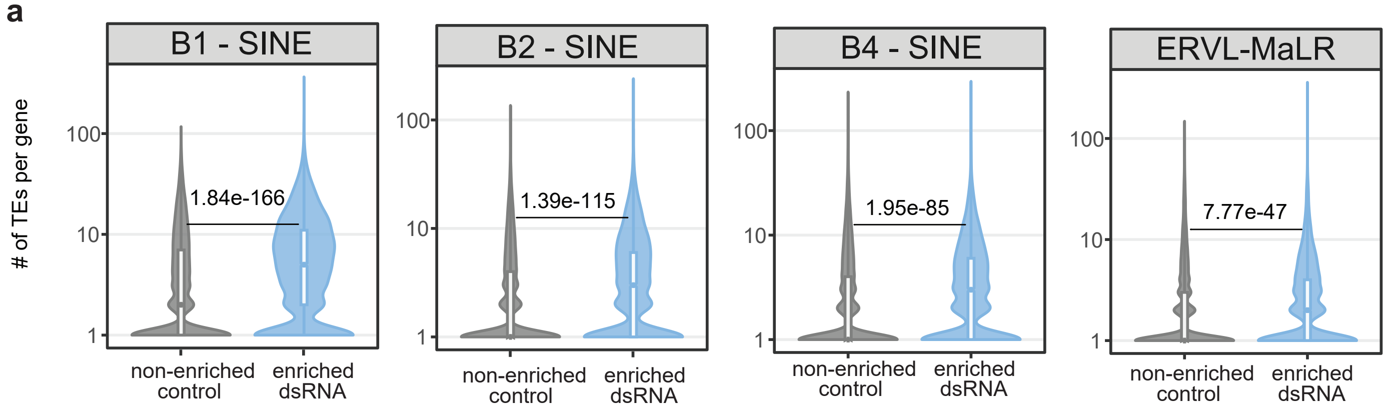

Supplementary Figure 5. TE content in dsRNA by families (**a**) Retrotransposon families enriched in the dsRNA IPs vs non-enriched control. These include the SINEs, B1, B2 and B4, as well as the LTR, ERV-MaLR.

Supplementary Figure 6

a

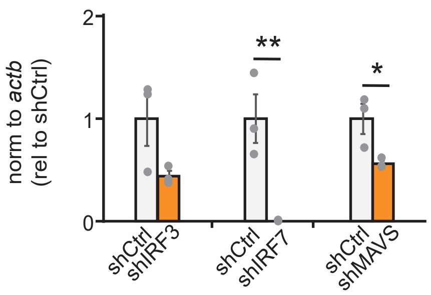

Supplementary Figure 6. shRNA depletion levels. (**a**) RT-qPCR analyses for depletion levels of *Irf3*, *Irf7* and *Mavs*. Data are the average of three biological replicates ± SEM, Single factor ANOVA was used to calculate significant differences amongst comparisons, followed by an F-test for variance and appropriate two-tailed t-test (*) p-val≤0.05, (**) p-val≤0.01, (***) p-val≤0.001.

Supplementary Figure 7

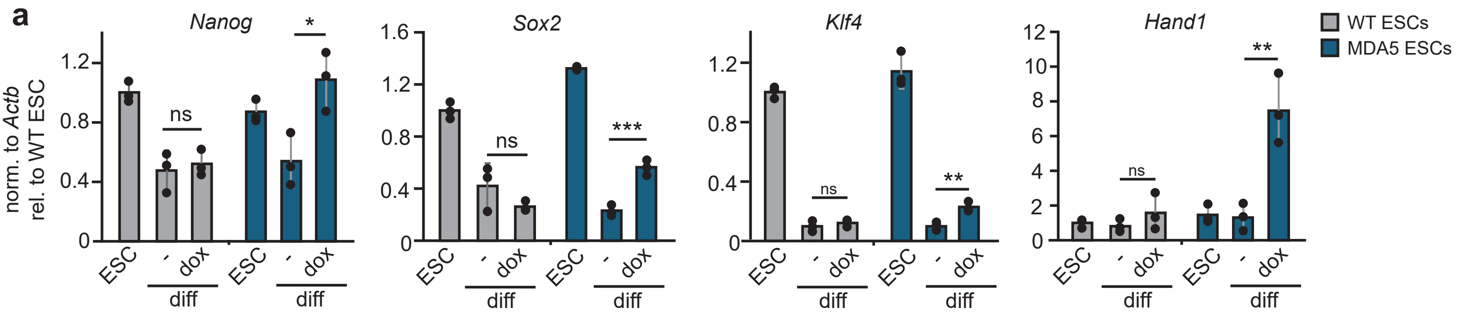

Supplementary Figure 7. Differentiation of mESCs is affected upon MDA5 and IFN activation (a) WT and MDA5 (clone 1) expressing ESCs were differentiated *in vitro* using embryoid bodies for 24 hours in the presence or absence of doxycycline. Expression of pluripotency *(Nanog, Sox2, Klf4*) and differentiation markers (*Hand1*) were compared in untreated cells (ESCs), differentiating cells without doxycycline (-), and differentiating cells in the presence of doxycycline (dox). Data represent the average of three biological replicates ± SD. Single factor ANOVA was used to calculate significant differences amongst comparisons, followed by an F-test for variance and appropriate two-tailed t-test, (*) p-val≤0.05, (**) p-val≤0.01, (***) p-val≤0.001.

Supplementary Figure 8.1

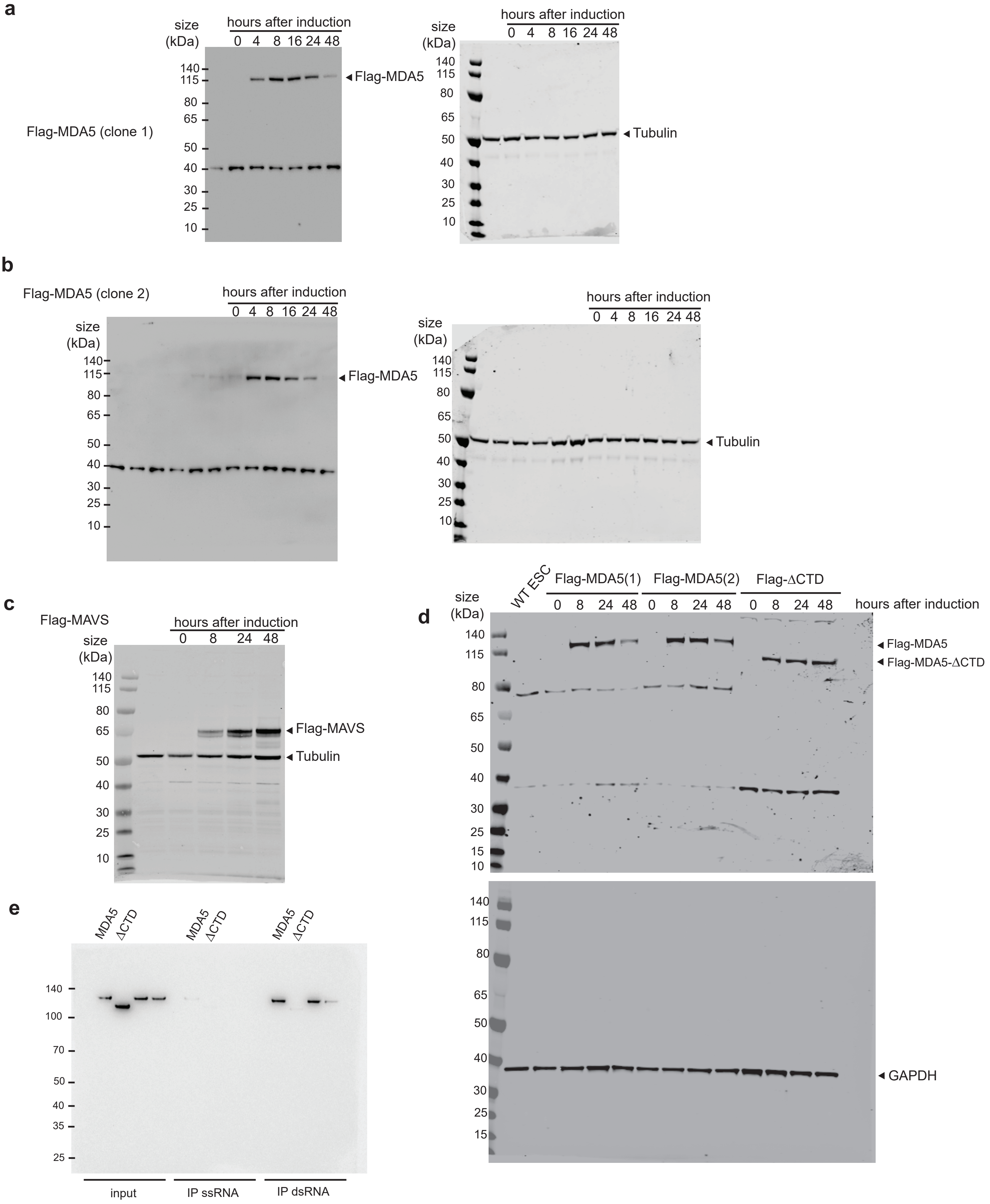

Supplementary Figure 8.2

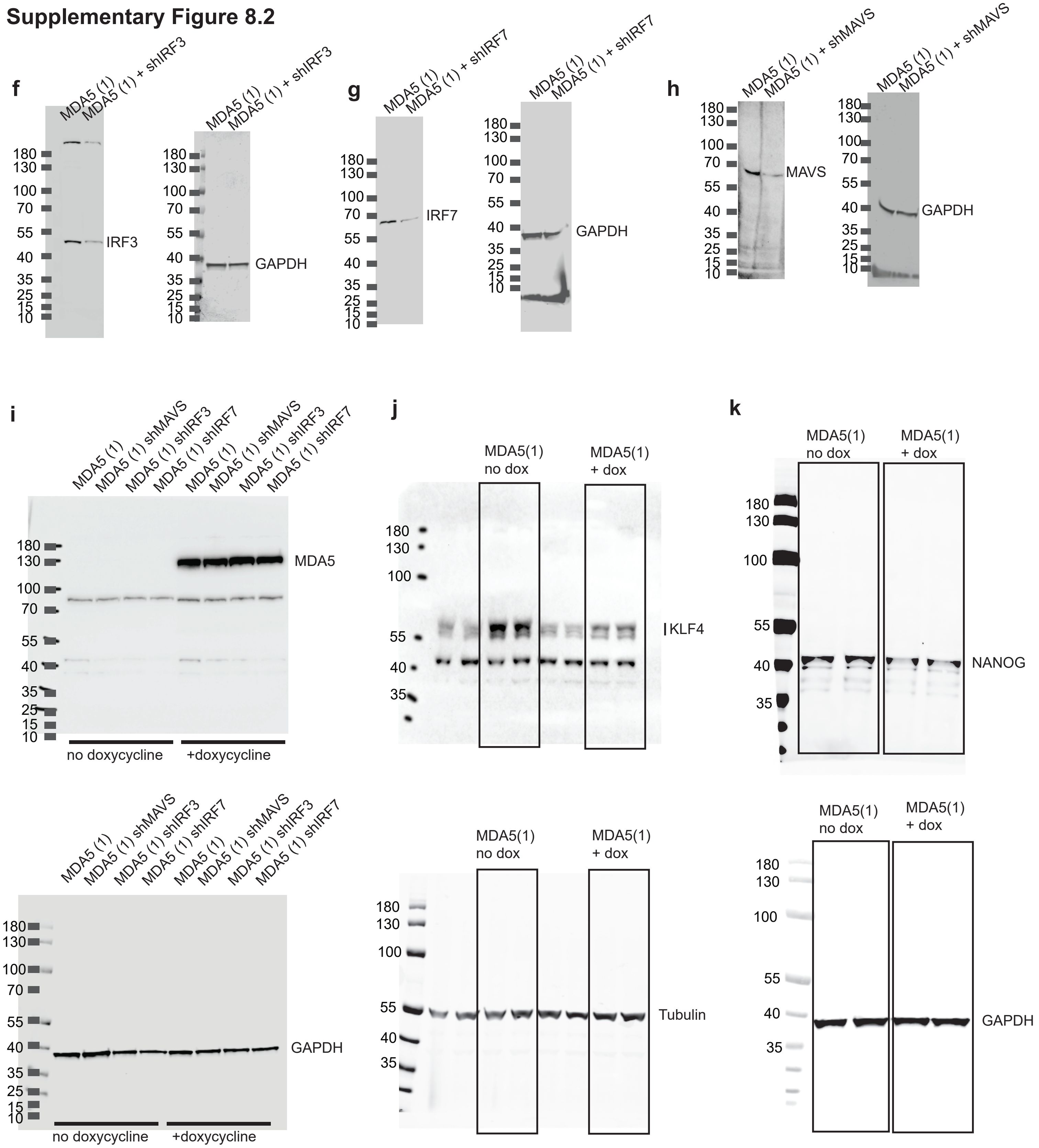

Supplementary Figure 8. Uncropped western blots. (**a**) FLAG-MDA5 clone 1 time course with anti-FLAG antibody (left) and Tubulin as loading control (right). Figure 2a in main text. (**b**) FLAG-MDA5 clone 2 time course with anti-FLAG antibody (left) and Tubulin as loading control (right). Figure 2a in main text. (**c**) FLAG-MAVS time course western blot with FLAG and Tubulin antibodies probed on the same blot. Figure 6e in main text. (**d**) (top) Time course of FLAG-tagged MDA5 clones (1) and (2) and ΔCTD mutant after 0, 8, 24, and 48 hours of doxycycline addition. Figure 4d in main text. (bottom) GAPDH blot was used as a loading control. (**e**) anti-FLAG western blot against purified FLAG-MDA5 WT and ΔCTD, in both input reactions and pull-downs with beads coated with ssRNA or dsRNA. Figure 4c in main text. (**f, g, h**) Western blot of analyses of MDA5(1) (clone 1) stably depleted with shRNAs against IRF3 (**f**), IRF7 (**g**), and MAVS (**h**). Figure 6a in main text. GAPDH serves as a loading control. (**i**) Western blot analyses of FLAG-MDA5 induction in cells depleted of IRF3, IRF7 and MAVS by shRNAs. GAPDH serves as a loading control (bottom). Uninduced (no dox) and induced (+dox) conditions were compared. Figure 6b in main text. (**j**) Western blot analysis of KLF4 protein levels in two biological replicates of uninduced MDA5(1) ESCs (no dox) vs two biological replicates of MDA5(1) cells treated with doxycycline for 14 hours (+dox). Tubulin serves as a loading control (bottom). Figure 3e in main text. (**k**) Western blot analysis of NANOG protein levels in two biological replicates of uninduced MDA5(1) ESCs (no dox) vs two biological replicates of MDA5(1) cells treated with doxycycline for 14 hours (+dox). GAPDH serves as a loading control (bottom). Figure 3e in main text.

Supplementary References

1. Sladitschek, H. L. & Neveu, P. A. A gene regulatory network controls the balance between mesendoderm and ectoderm at pluripotency exit. Mol. Syst. Biol. 15, e9043 (2019).

2. Maslon, M. M. et al. A slow transcription rate causes embryonic lethality and perturbs kinetic coupling of neuronal genes. EMBO J. 38, e101244 (2019).

3. Bonev, B. *et al.* Multiscale 3D Genome Rewiring during Mouse Neural Development. *Cell* **171**, 557-572.e24 (2017).

4. Dobrinić, P., Szczurek, A. T. & Klose, R. J. PRC1 drives Polycomb-mediated gene repression by controlling transcription initiation and burst frequency. *Nat. Struct. Mol. Biol.* **28**, 811–824 (2021).
