# Supplementary material for: Double stranded RNA sensing is silenced during early embryonic development
Source: Nat Commun. 2025 Dec 11;16:11438. doi: 10.1038/s41467-025-66352-0 (PMC12749073; doi:10.1038/s41467-025-66352-0)
Supplement: Supplementary file 2 — Reporting Summary [file 41467_2025_66352_MOESM2_ESM.pdf]

nature portfolio

Corresponding author(s): Dr. Sara Macias
smacias@ed.ac.uk

Last updated by author(s): Oct 6, 2025

Reporting Summary

Nature Portfolio wishes to improve the reproducibility of the work that we publish. This form provides structure for consistency and transparency in reporting. For further information on Nature Portfolio policies, see our Editorial Policies and the Editorial Policy Checklist.

Statistics

For all statistical analyses, confirm that the following items are present in the figure legend, table legend, main text, or Methods section.

n/a	Confirmed	
☐	☒	The exact sample size (n) for each experimental group/condition, given as a discrete number and unit of measurement
☐	☒	A statement on whether measurements were taken from distinct samples or whether the same sample was measured repeatedly
☐	☒	The statistical test(s) used AND whether they are one- or two-sided<br>*Only common tests should be described solely by name; describe more complex techniques in the Methods section.*
☒	☐	A description of all covariates tested
☐	☒	A description of any assumptions or corrections, such as tests of normality and adjustment for multiple comparisons
☐	☒	A full description of the statistical parameters including central tendency (e.g. means) or other basic estimates (e.g. regression coefficient) AND variation (e.g. standard deviation) or associated estimates of uncertainty (e.g. confidence intervals)
☐	☒	For null hypothesis testing, the test statistic (e.g. F, t, r) with confidence intervals, effect sizes, degrees of freedom and P value noted<br>*Give P values as exact values whenever suitable.*
☒	☐	For Bayesian analysis, information on the choice of priors and Markov chain Monte Carlo settings
☒	☐	For hierarchical and complex designs, identification of the appropriate level for tests and full reporting of outcomes
☒	☐	Estimates of effect sizes (e.g. Cohen's d, Pearson's r), indicating how they were calculated

Our web collection on statistics for biologists contains articles on many of the points above.

Software and code

Policy information about availability of computer code

Data collection	StepOne software v2.3<br>Quantstudio Design&Analysis v1.5.2<br>Azure Cielo manager v1.0.8.28<br>MaxQuant analyzer 10
Data analysis	StepOne Software v2.3<br>Quantstudio Design&Analysis v1.5.2<br>Azure Cielo manager v1.0.8.28<br>ImageJ v1.51p<br>ImageQuant TL<br>Microsoft Excel<br>Cytoscape v3.10.3<br>ClueGo v2.5.10<br>CutAdapt v3.4<br>Hisat2 v2.2.1<br>FeatureCounts<br>DESeq2<br>FlowJo<br>Enrichr<br>ShinyGO v0.81<br>MaxQuant v2.4.2

Perseus v2.0.11
R v4.4.1
Fastp v0.32.2
Genomepy 0.16.1
MarkDuplicates v3.0.0
macs2 v2.2.7
Diffbind v3.12.0
Deeptools v3.5.5
ChIPseeker v1.38.0
Clusterprofiler v4.10.1
EdgeR v4.2.2
ViennaRNA v2.7.0
GenomicRanges v1.56.2

For manuscripts utilizing custom algorithms or software that are central to the research but not yet described in published literature, software must be made available to editors and reviewers. We strongly encourage code deposition in a community repository (e.g. GitHub). See the Nature Portfolio guidelines for submitting code & software for further information.

Data

Policy information about availability of data

All manuscripts must include a data availability statement. This statement should provide the following information, where applicable:

- Accession codes, unique identifiers, or web links for publicly available datasets
- A description of any restrictions on data availability
- For clinical datasets or third party data, please ensure that the statement adheres to our policy

Source data are provided with this paper. The RNAseq data of MDA5 induction, dsRNA IP and ATACseq have been deposited on SRA under accession numbers PRJNA1219136 https://www.ncbi.nlm.nih.gov/bioproject/PRJNA1219136, PRJNA1223341 https://www.ncbi.nlm.nih.gov/bioproject/PRJNA1223341 and PRJNA1224926 https://www.ncbi.nlm.nih.gov/bioproject/PRJNA1224926, respectively. The proteomics data has been deposited in the PRIDE database under accession number PXD059977 https://www.ebi.ac.uk/pride/archive/projects/PXD059977.
Existing datasets used in this study include the mouse ESC differentiation datasets E-MTAB-4904 https://www.ebi.ac.uk/biostudies/ArrayExpress/studies/E-MTAB-4904 and GSE127741 https://www.ncbi.nlm.nih.gov/geo/query/acc.cgi?acc=GSE127741. And zebrafish dataset GSE106430 https://www.ncbi.nlm.nih.gov/bioproject/PRJNA416866.

Research involving human participants, their data, or biological material

Policy information about studies with human participants or human data. See also policy information about sex, gender (identity/presentation), and sexual orientation and race, ethnicity and racism.

Reporting on sex and gender	No human participants, their data, or biological material were involved in this study
Reporting on race, ethnicity, or other socially relevant groupings	No human participants, their data, or biological material were involved in this study
Population characteristics	see above
Recruitment	no participant were recruited
Ethics oversight	No human participants, their data, or biological material were involved in this study

Note that full information on the approval of the study protocol must also be provided in the manuscript.

Field-specific reporting

Please select the one below that is the best fit for your research. If you are not sure, read the appropriate sections before making your selection.

☒ Life sciences ☐ Behavioural & social sciences ☐ Ecological, evolutionary & environmental sciences

For a reference copy of the document with all sections, see nature.com/documents/nr-reporting-summary-flat.pdf

Life sciences study design

All studies must disclose on these points even when the disclosure is negative.

Sample size	Sample sizes were based on variation observed in pilot experiments and the author's experience. For proteomics and ATACseq experiments expert advice was sought on sample size. Statistical analysis of the experiments confirmed that the sample size was adequate.

Data exclusions	Data were only excluded in case of user error
Replication	At least three (n=3 or more) biological replicates for all experiments were performed. Only ChEP-MS and ATAC-seq were n=2, as reproducibility can be achieved based on previous experience. Aside from user error, attempts at replication were successful.
Randomization	Randomizations were not necessary for the type of experiments presented in this study
Blinding	No blinding was necessary in this study as almost all data was quantitative and measured by machines. In cases where a certain level of subjectivity in observations might be present, the results were always verified by quantitative, machine derived data.

Reporting for specific materials, systems and methods

We require information from authors about some types of materials, experimental systems and methods used in many studies. Here, indicate whether each material, system or method listed is relevant to your study. If you are not sure if a list item applies to your research, read the appropriate section before selecting a response.

Materials & experimental systems

n/a	Involved in the study
☐	☒ Antibodies
☐	☒ Eukaryotic cell lines
☒	☐ Palaeontology and archaeology
☐	☒ Animals and other organisms
☒	☐ Clinical data
☒	☐ Dual use research of concern
☒	☐ Plants

Methods

n/a	Involved in the study
☒	☐ ChIP-seq
☐	☒ Flow cytometry
☒	☐ MRI-based neuroimaging

Antibodies

Antibodies used	J2 (English & Scientific Consulting) 1:400 (IF)<br>Anti-FLAG (M2, Merck) 1:4000 (WB)<br>Anti-Tubulin (CP06, Calbiochem) 1:5000 (WB)<br>Anti-GAPDH (CB1001, Merck) 1:5000 (WB)<br>Anti-Nanog (ebioMLC-51, Invitrogen) 1:1000 (WB)<br>Anti-KLF4 (4038, CST) 1:1000 (WB)<br>Anti-IRF3 (12A4A35, BioLegend) 1:1000 (WB)<br>Anti-IRF7 (MA5-52511, Invitrogen) 1:1000 (WB)<br>Anti-MAVS (sc365334, Santa Cruz). 1:1000 (WB)<br>Anti-mouse FITC (406001, Biolegend) 1:2000 (IF)<br>anti-mouse HRP (7076, CST) 1:1000 (WB)<br>anti-Rabbit HRP (7074, CST) 1:1000 (WB)<br>anti-mouse 680RD (925-68070, LICORbio) 1:10000 (WB)<br>anti-mouse 800CW (926-32210, LICORbio) 1:10000 (WB)<br>anti-rabbit 680RD (925-68071, LICORbio) 1:10000 (WB)<br>anti-rabbit 800CW (926-32211, LICORbio) 1:10000 (WB)
Validation	Anti-FLAG was validated by the authors by overexpressing FLAG proteins in eukaryotic cells and testing these with the antibody α-tubulin was validated by the manufacturer (http://www.merckmillipore.com/GB/en/product/Anti-Tubulin-Mouse-mAb-DM1A,EMD_BIO-CP06) and externally (Immunoblotting Gagarin, D., et al. 2005. J. Mol. Cell. Card. 39, 453. Yang, Z., et al. 2007. J. Vasc. Res. 44, 483). The rest of the antibodies have been successfully used by other publications. For pluripotency markers, we have used differentiated cells as negative controls. For MAVS, IRF3 and IRF7 antibodies, we have confirmed their specificity after depletion of the indicated proteins.<br>J2 has been extensively used in numerous publications. Furthermore, as a control, we used a dsRNA targeting nuclease (RNase III) to deplete cells from dsRNAs and consequent lose antibody signal.

Eukaryotic cell lines

Policy information about cell lines and Sex and Gender in Research

Cell line source(s)	v6.5 purchased from ThermoFisher (MES1402)<br>BV-2 were kindly donated by Prof. P. Simmonds<br>HEK293T were donated by Prof. Javier F. Caceres
Authentication	Morphological appearance, qRT-PCR of pluripotency and differentiation-factor levels were used to validate the mouse embryonic stem cell line v6.5.<br>Besides its distinctive morphological appearance, no additional authentication methods were used for the BV-2 or HEK293T cell line.

Mycoplasma contamination	Regular testing were all negative
Commonly misidentified lines (See ICLAC register)	No commonly misidentified lines were used in this study

Animals and other research organisms

Policy information about studies involving animals; ARRIVE guidelines recommended for reporting animal research, and Sex and Gender in Research

Laboratory animals	Zebrafish wild-type strains AB/Tübingen/TAB (AB/Tu/TAB)
Wild animals	This study did not involve wild animals
Reporting on sex	This information has not been collected
Field-collected samples	Study did not involve samples collect from the field
Ethics oversight	All experiments performed with zebrafish at the University of Granada comply with national and European Community regulations for the use of animals in experimentation and were approved by the ethical committees of the University of Granada and the Junta de Andalucía.

Note that full information on the approval of the study protocol must also be provided in the manuscript.

Plants

Seed stocks	No plant materials were used
Novel plant genotypes	No plant materials were used
Authentication	No plant materials were used

Flow Cytometry

Plots

Confirm that:

☒ The axis labels state the marker and fluorochrome used (e.g. CD4-FITC).

☒ The axis scales are clearly visible. Include numbers along axes only for bottom left plot of group (a 'group' is an analysis of identical markers).

☒ All plots are contour plots with outliers or pseudocolor plots.

☒ A numerical value for number of cells or percentage (with statistics) is provided.

Methodology

Sample preparation	Cells were dissociated using 0.05% Trypsin, washed in PBS and resuspended in FACS buffer (PBS with 1% FBS). Cells were pelleted and resuspended in Fixation buffer (420801, BioLegend) and incubated for 15’ at 4°C, washed twice with Intracellular Staining Permeabilisation Wash Buffer (421002, BioLegend) and stained overnight with the anti-dsRNA antibody J2 (English & Scientific Consulting) in Intracellular Staining Permeabilisation Wash Buffer. After washing, cells were incubated with anti-mouse Alexa Fluor 647 (ThermoFisher) for one hour at room temperature and washed three times with Intracellular Staining Permeabilisation Wash Buffer before finally resuspending the cells in FACS buffer.
Instrument	MACS Quant analyzer 10 (Miltenyi)
Software	FlowJo
Cell population abundance	After gating of the total cell population (FSC-SSC) which depending on the cell line was 70-90% of the total counted events, singlets were selected which were at least 95% of the cells. The number of cells identified as positive depended on the cell line an treatment and varied between 0.6 and 1% for the negative control to 99.1% for the mock controls.
Gating strategy	After gating of the total cell population (FSC-SSC) for the relevant fraction of cells, singlets were selected using FSC-H/FSC-A

Gating strategy

and gating for the main upper population. These singlets were selected and a negative gate was selected on the 'secondary only' control cells. Cells were considered positive if the signal was higher than this negative gate.

☒ Tick this box to confirm that a figure exemplifying the gating strategy is provided in the Supplementary Information.
