# Supplementary material for: Double stranded RNA sensing is silenced during early embryonic development
Source: Nat Commun. 2025 Dec 11;16:11438. doi: 10.1038/s41467-025-66352-0 (PMC12749073; doi:10.1038/s41467-025-66352-0)
Supplement: Supplementary file 3 — Description of Additional Supplementary Files [file 41467_2025_66352_MOESM3_ESM.pdf]

Description of Additional Supplementary Files

File name: ***Supplementary data 1 - RNAseq - log2 normalised counts.xlsx***

Description: Log2 normalised counts (Deseq2) of RNAseq of v6.5, v6.5 - MDA5(1) and v6.5 - MDA5(2) for time points 0,4,8 and 24. Three biological replicates for each cell line and each time point.

File name: ***Supplementary data 2 - RNAseq - shared genes two clones.xlsx***

Description: Upregulated and downregulated genes (relative to WT) that are shared between clones v6.5-MDA5(1) and v6.5-MDA5(2) for time points 4, 8 and 24hours after induction of MDA5 expression.

File name: ***Supplementary data 3 - ATACseq - differential peaks.xlsx***

Description: List of peaks with significantly increased and decreased accessibility 24 hours post induction compared to WT.

File name: ***Supplementary data 4 - ChEP-MS - data_LFQ.xlsx***

Description: ChEP-MS data of proteins detected (LFQ) for WT and MDA5(1) for time points 0, 8, 24 and 48

File name: ***Supplementary data 5 - oligonucleotides.xlsx***

Description: List of oligos used for RT-qPCR, construction of shRNA plasmid used to make stable cell lines, Gibson assembly oligos for constructing the 3xFLAG-MDA5, 3xFLAG-MDA5dCTD and MAVS plasmid and consequent stable cell lines.
